# The Quest for a Vaccine That Yields Tumor-Killing T cells

**DOI:** 10.1371/journal.pmed.0010050

**Published:** 2004-11-30

The immune system has a remarkable capacity for fending off infectious diseases, and it has become clear that these same defenses can recognize and destroy cancer cells. In fact, they do so on an ongoing basis, and cancer develops only when immune surveillance breaks down. Many patients with established tumors also mount an immune response against some antigens that are specific to, or enriched in, the tumor. This response, however, is rarely effective against the disease.

The idea of enlisting the immune system to fight cancer has been around for a long time, and has led to the development of various cancer vaccines designed to alert the immune system to the presence of a tumor and to induce a response that, selectively and potently, will eliminate tumor cells. Vaccines include whole tumor extracts or specific proteins and peptides that are selectively expressed or enriched in tumors, by themselves or with a variety of adjuvants.

There have been some spectacular successes, in particular with immune therapy to malignant melanoma, a tumor type that seems naturally to be more immunogenic than others. However, even in melanoma, success is usually restricted to a fraction of the patients, with no obvious explanation of why the strategy works for a particular patient and fails in most others. The emphasis has consequently shifted from clinical outcomes to monitoring a patient's immune response. What type of response is necessary and sufficient to eliminate tumor cells is still unclear, but the hope is that understanding the immune response in patients that show clinical benefit will answer that question.

Peter Lee and colleagues used state-of-the art technology to dissect the endogenous immune response to vaccination with heteroclitic melanoma peptides, i.e., melanoma-associated peptides that have been engineered to elicit a stronger immune response. They focused on cytotoxic T lymphocytes (CTLs), and compared CTL clones from four melanoma patients who had vaccine-induced T cell responses and two melanoma patients with spontaneous anti-tumor T cell responses. The researchers analyzed several hundred CTL clones (to get a sense for the complexity of the responses in individual patients) for T cell receptor variable chain beta expression, recognition efficiency, and ability to lyse target melanoma cells. Most T cells isolated from vaccinated patients were poor at tumor cell lysis compared with T cells from endogenous responses to cancer.[Fig pmed-0010050-g001]

**Figure pmed-0010050-g001:**
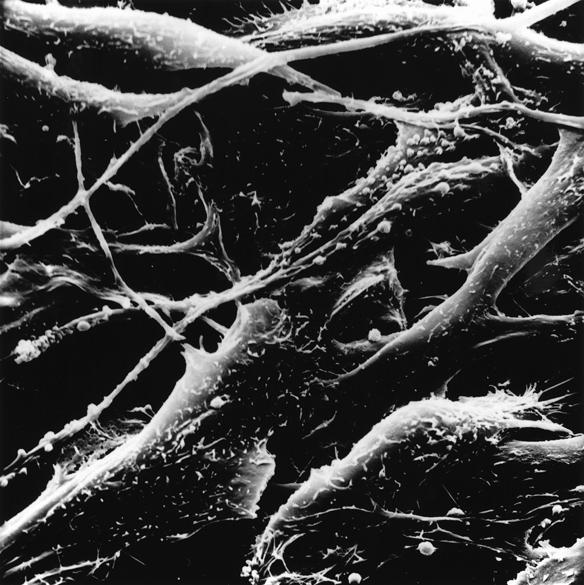
Melanoma—a prime target of cancer vaccines (Photo: Timothy Triche, National Cancer Institute)

The authors suggest that the high doses of peptides administered in vaccinations and the increased binding capacity of heteroclitic peptides to major histocompatibility complex molecules—the very quality that makes them more immunogenic—induce many T cells with low recognition efficiency for the native peptides they encounter on the tumor cells. Their findings also bring into question the ability to deduce the recognition efficiency and tumor reactivity of T cell responses from ELISPOT and tetramer staining assays—the two standard measures of T cell responses to vaccines—which has implications for rational vaccine design in general.

